# Discovery of a novel dominant mutation in the *REN* gene after forty years of renal disease: a case report

**DOI:** 10.1186/s12882-017-0631-5

**Published:** 2017-07-12

**Authors:** Rhian L. Clissold, Helen C. Clarke, Olivera Spasic-Boskovic, Kim Brugger, Stephen Abbs, Coralie Bingham, Charles Shaw-Smith

**Affiliations:** 1 Royal Devon and Exeter NHS Foundation Trust, Barrack Road, Exeter, Devon EX2 5DW UK; 2 University of Exeter Medical School, Barrack Road, Exeter, Devon EX2 5DW UK; 3 Department of Clinical Genetics, Cambridge University Hospitals NHS Foundation Trust, Cambridge, UK

**Keywords:** Kidney disease, Genetics, Renin, Case report

## Abstract

**Background:**

Heterozygous mutations in the gene encoding renin (*REN*) cause autosomal dominant tubulointerstitial kidney disease (ADTKD), early-onset anaemia and hyperuricaemia; only four different mutations have been described in the published literature to date. We report a novel dominant *REN* mutation discovered in an individual after forty years of renal disease.

**Case presentation:**

A 57 year old Caucasian woman with chronic kidney disease stage five was reviewed in a regional joint renal genetics clinic. She had initially been diagnosed with chronic pyelonephritis in adolescence, around the same time that she was investigated for anaemia out of keeping with her degree of renal impairment. Hyperuricaemia was identified in her twenties following an episode of gout. A diagnosis of ADTKD was not made until the age of 37 years, when her mother was also found to have kidney disease and commenced haemodialysis. The patient’s renal function continued to slowly deteriorate and, twenty years later, her sister was worked up as a potential donor for kidney transplantation. Revisiting the maternal family history during the transplant work up prompted a referral to clinical genetics and urgent *REN* genetic testing was requested for the patient, leading to discovery of a heterozygous mutation in the *REN* gene: c.49 T > C, p.(Trp17Arg). This variant was not identified in her otherwise healthy sister, allowing pre-emptive live renal transplantation to take place shortly afterwards.

**Conclusions:**

In an era where genetic testing is becoming much more readily available, this case highlights the importance of considering a genetic aetiology in all patients with long-standing renal disease and a relevant family history. Establishing a genetic diagnosis of ADTKD-*REN* in this individual with chronic anaemia, hyperuricaemia and slowly progressive renal impairment helped to identify a suitable live kidney donor and allowed successful pre-emptive transplantation to take place.

## Background

Heterozygous mutations in the genes encoding uromodulin (*UMOD*), [1] hepatocyte nuclear factor 1β (*HNF1B*), [2] renin (*REN*) [3] and mucin-1 (*MUC1*) [4] can result in autosomal dominant tubulointerstitial kidney disease (ADTKD). This has been known by several other terms in the past, most frequently medullary cystic kidney disease. ADTKD is characterised by progressive tubulointerstitial fibrosis and development of end-stage renal disease (ESRD). [5] Other clinical features include a positive family history in the majority of cases, bland urinary sediment with little or no proteinuria, normal or small-sized kidneys on renal imaging and normotension during the early stages of the disease. [6–8] The prevalence is unknown; however, it is likely that many cases remain undetected given the lack of standardised diagnostic criteria and variable terminology prior to the recent Kidney Disease: Improving Global Outcomes consensus report. [5] As genetic testing for the genes known to be associated with ADTKD becomes more widely available in routine clinical practice, it will be important to increase awareness of the condition amongst nephrologists and other health care professionals who may have affected patients without a genetic diagnosis.

Certain factors can be helpful in differentiating the various subtypes of ADTKD. ADTKD-*UMOD* is classically associated with hyperuricaemia and gout, secondary to a decreased fractional excretion of urate. [8] ADTKD-*HNF1B* may be accompanied by a variety of extra-renal phenotypes, including diabetes mellitus, genital tract malformations and abnormal liver function. [9] The hallmark of ADTKD-*REN* is early-onset anaemia out of proportion to the level of kidney failure. [3] Other findings in patients with *REN* mutations include mild hypotension, an increased risk of acute kidney injury (AKI), hyperuricaemia and hyperkalaemia [5].

**Fig. 1:**
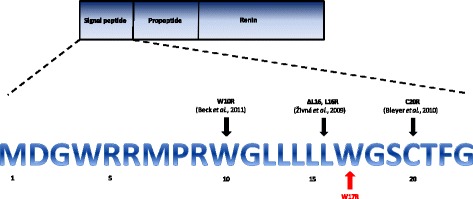
Schematic of the *REN* gene; the amino acid sequence of the signal peptide is shown below with the locations of the published mutations (indicated by the *black arrows*) and novel mutation described in this case report (indicated by the *red arrow*) identified

*REN* is located on chromosome 1 and is mainly expressed in granular cells in the juxtaglomerular apparatus. It encodes preprorenin, which contains a signal sequence that targets it to the endoplasmic reticulum for glycosylation and proteolytic processing to produce prorenin and subsequently renin. [10] Renin is a protease that cleaves angiotensinogen to angiotensin and stimulates aldosterone production. Mutations in the *REN* gene lead to intracellular accumulation of abnormal protein, resulting in apoptosis of renin-producing cells in the juxtaglomerular apparatus with consequent nephron loss and progressive chronic kidney disease (CKD). [3] To date, only four different heterozygous *REN* mutations have been reported in the published literature (Fig. 1). [3, 11, 12] All are localised to exon 1, which encodes the signal sequence. In this case report, we describe a novel dominant *REN* mutation discovered as part of a transplant work up and aim to highlight the importance of investigating a genetic diagnosis in patients with long-standing renal disease and a relevant family history.

## Case presentation

**Fig. 2:**
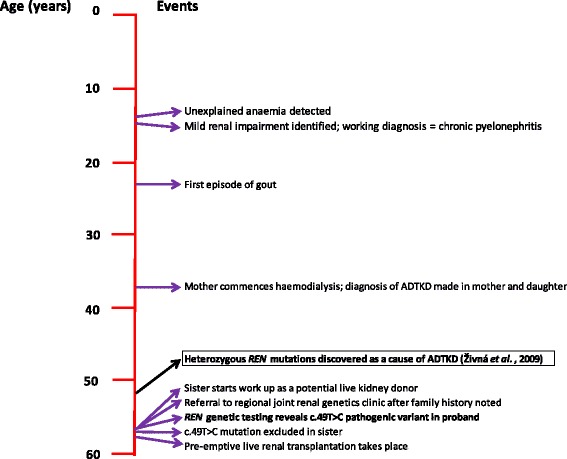
Timeline showing significant events in the course of the proband’s renal disease that led to the eventual diagnosis of ADTKD-*REN*. Abbreviations: ADTKD, autosomal dominant tubulointerstitial kidney disease

A 57 year old White British woman with CKD stage five was referred to the regional joint renal genetics clinic after her sister was noted to have a family history of renal disease during work up as a potential live kidney donor. The patient had been under nephrological follow up since adolescence, when she was incidentally discovered to have mild renal impairment (Fig. 2). An intravenous pyelogram performed at the time showed that both kidneys were small with decreased cortical thickness and the most likely diagnosis was felt to be chronic pyelonephritis. Ultrasound scanning confirmed small kidneys with no overt renal cysts observed; urinalysis was consistently bland. She had also been investigated for symptoms of marked tiredness as a teenager and was found to be anaemic with a haemoglobin value of approximately 90 g/L. This was felt to be out of keeping with her level of kidney function at the time and remained unexplained despite extensive investigation. She developed gout in her twenties and her serum uric acid level was measured at 550 μmol/L; treatment with allopurinol was initiated. She remained under renal follow up and her kidney function continued to slowly deteriorate. Her haemoglobin level remained stable at around 100 g/L after initial treatment with iron and later an erythropoiesis-stimulating agent (ESA). When she was aged 37 years, one of the nephrology team noted that her mother had recently commenced haemodialysis. As both mother and daughter had presented with slowly progressive CKD, bland urinary sediment and small kidneys on renal imaging, the most likely diagnosis was now felt to be ADTKD.

**Fig. 3:**
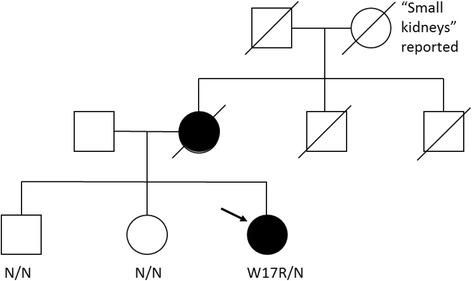
Pedigree of reported family. The *REN* phenotype is indicated below the relevant symbol where known. *Black* symbols denote affected individuals with confirmed renal disease. The proband is marked with an *arrow*

A detailed family history revealed that the patient’s maternal grandmother had been told she had small kidneys but no further clinical details were available (Fig. 3). The patient’s mother was identified as having mild CKD and hypertension at the age of 65 years during a routine health screen; renal ultrasound scanning at the time showed two small kidneys. Her renal function continued to decline and she commenced haemodialysis at the age of 70 years, before passing away 5 years later due to infective endocarditis. The patient’s siblings were all reported as being healthy.

**Fig. 4:**
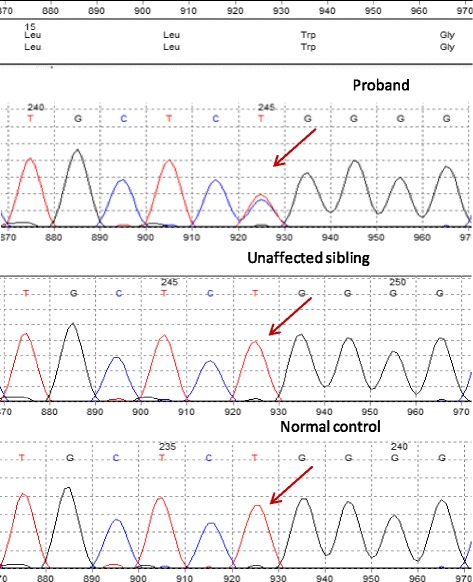
Sanger sequencing traces for the pathogenic *REN* variant, c.49 T > C: present in proband (*upper panel*) and absent in unaffected sibling and random normal control (*middle* and* lower panels*, respectively)

By the time the patient was reviewed in the renal genetics clinic, her estimated glomerular filtration rate had fallen to 12 and she was hoping to undergo imminent pre-emptive live renal transplantation with her sister as the donor. Given her clinical diagnosis of ADTKD, hyperuricaemia and history of young-onset anaemia, urgent next generation sequencing was performed using the Illumina TruSight One sequencing kit and the data was analysed to search for pathogenic variants in *HNF1B* (NM_000458.2), *REN* (NM_000537.3) and *UMOD* (NM_003361.2). The full coding regions (+/−five base pairs) of all three genes were sequenced to a depth of 20 fold or more, with analytical sensitivity of 98.3%–100% (95% confidence intervals). This identified a heterozygous and likely pathogenic variant in the *REN* gene: c.49 T > C, p.(Trp17Arg); no abnormalities were detected in *HNF1B* or *UMOD* (Fig. 4). Although this sequence change has not previously been reported in the published literature, missense pathogenic mutations in the same region of *REN* have been described and associated with a similar phenotype. [3, 11, 12] The c.49 T > C variant alters a highly conserved nucleotide and weakly conserved amino acid in the aspartic peptidase domain of renin. Although in silico analyses are inconsistent and classify the variant from tolerated to possibly damaging, it has not been reported in the Exome Aggregation Consortium, Exome Sequencing Project or 1000 Genomes Project and is therefore very rare. [13–15] Functional studies have shown that a mutation in the adjacent amino acid, p.(Leu16Arg), prevents prorenin and renin biosynthesis and secretion. [3] Therefore, it was considered highly likely that the *REN*: c.49 T > C, p.(Trp17Arg) variant was the cause of disease in this patient. Unpublished observations presented by Vincent Morinière and colleagues at the 43rd Annual Meeting and Scientific Exposition of the American Society of Nephrology in 2010 include discovery of the same p.(Trp17Arg) *REN* mutation in an individual with chronic tubulointerstitial nephritis and hyperuricaemia with a family history of both tubulointerstitial kidney disease and gout, providing further evidence for the pathogenicity of this variant.

The patient’s sister commenced work up as a live kidney donor aged 53 years. She was normotensive with bland urinalysis and a serum creatinine of 72 μmol/L. Her serum urate level was also within normal limits at 250 μmol/L. Renal ultrasound scanning suggested a slight size discrepancy between the right and left kidneys of approximately 1.6 cm; she went on to have magnetic resonance angiography of the kidney, which showed no evidence of narrowing or dilatation of the renal arteries. Urgent *REN* genetic testing excluded the c.49 T > C pathogenic variant identified in her sister (Fig. 4). This allowed pre-emptive live donor transplantation with a 0:0:0 HLA mismatch to take place shortly afterwards. The patient’s serum creatinine was 124 μmol/L one month post-transplant and 139 μmol/L when last checked; her haemoglobin levels have remained approximately 100 g/L despite stopping treatment with an ESA at the time of the transplant.

## Discussion

This case highlights the importance of revisiting an individual’s clinical diagnosis each time a new piece of medical information becomes available. Mild renal impairment and unexplained anaemia were detected in our patient during her teenage years. Young-onset anaemia disproportionate to the degree of renal impairment is now known to be associated with ADTKD-*REN*. Haemoglobin levels tend to normalise during puberty; the reason for this remains unclear, although increased testosterone secretion after adolescence in males has been hypothesised as a possible cause. [3] She suffered her first episode of gout in her twenties and was found to be hyperuricaemic. Early-onset gout in a young female with mild CKD provided an early clue to the later diagnosis of ADTKD. Raised serum urate levels in individuals with *REN* mutations are likely due to increased reabsorption of urate in the proximal tubule. The production of normal renin is significantly reduced, resulting in low renin and aldosterone levels and subsequently mild hypotension and hyperkalaemia. This relative hypotension stimulates the reabsorption of sodium in the proximal tubule, which consequently leads to an increase in urate reabsorption and hyperuricaemia. [3, 11].

Discovery of the maternal family history of renal disease in this case led to the clinical diagnosis of ADTKD in our patient approximately 20 years after her renal impairment was first identified in the 1970s. Reviewing the family history again 20 years later as part of her sister’s live kidney donor work up led to the genetic diagnosis of ADTKD-*REN* in 2015. Heterozygous mutations in the *REN* gene were first recognised as a cause of ADTKD in 2009 and genetic testing is available as part of routine clinical practice in the United Kingdom despite only a handful of cases being described so far. [3, 11, 12] Several renal genetics clinics have been set up over the past decade in line with major advances in DNA sequencing technology and a rapidly expanding knowledge base; the first joint clinic covering Devon and Cornwall started in 2012. Establishing a genetic diagnosis has multiple advantages for individuals with renal disease and their families. Genetic testing may identify a condition with specific therapeutic options and can prevent the need for renal biopsy in affected family members. It also has an important role in the assessment of potential kidney donors within the family, as demonstrated in this case report. More accurate information about individual prognosis can be given when the genetic diagnosis is known and the option of both genetic counselling and pre-implantation genetic diagnosis can be discussed. It can also lead to participation in research projects and clinical trials. Finally, the therapeutic power of knowledge after potentially many years without a definite diagnosis cannot be underestimated; this is particularly relevant in a condition like ADTKD where many of the clinical and histological findings are often non-specific. The patient’s perspective of this case report highlights many of these points: *“It has been hugely beneficial to me to be able to undergo genetic testing and to have finally been given a diagnosis after all these years of suffering renal impairment. There have been numerous tests carried out over the years and each time I was told there was no conclusion that anybody could come to that would determine the cause of my renal failure, which I found hugely frustrating. Because of the genetic testing now available, my sister has been able to donate a kidney and this has completely transformed my life. Without her undergoing this test, it may not have been possible for her to go on to donate. This has also been extremely valuable for the rest of the family. My brother has been tested and found not to carry the mutation so subsequently his children are not affected either, which has brought great relief and peace of mind to them all. Other members of the family are currently being tested.”*

There is no randomised controlled trial data available for ADTKD. [5] Fludrocortisone has been used successfully in one ten-year-old patient with ADTKD-*REN* to increase both blood pressure and estimated glomerular filtration rate. Fludrocortisone should decrease abnormal renin production via feedback inhibition; this in turn should lead to less cell injury and may be beneficial in preserving renal function in the long term. [11] In terms of general treatment, patients with *REN* mutations should be managed according to established CKD guidelines. [16] However, they have an increased risk of volume depletion and AKI due to the renin-angiotensin system dysfunction that is seen. A low-salt diet, which is often recommended in CKD, should be avoided as it may exacerbate volume depletion. [5] Diuretics can worsen both hyperuricaemia and volume depletion so should be used with caution. [17] Individuals with ADTKD-*REN* are also likely to be particularly vulnerable to the acute decline in renal impairment that can be seen with nonsteroidal anti-inflammatory drugs. [11] Anaemia can be treated with erythropoiesis-stimulating agents. [3] As in this case, renal transplantation is the treatment of choice for ESRD as there is no disease recurrence in the graft. [5] Unlike for adult-onset disorders, children at risk of inheriting a *REN* mutation should be referred to a paediatric nephrology team as the disease frequently presents during childhood and they are likely to benefit from early management.

## Conclusions

In summary, we describe a novel dominant mutation in the *REN* gene in an individual with long-standing anaemia, hyperuricaemia and slowly progressive CKD. This case highlights the importance of revisiting the possibility of an underlying genetic cause in patients with long-standing renal disease and a relevant family history. Establishing a genetic diagnosis of ADTKD-*REN* in this individual after 40 years of renal disease helped to identify a suitable kidney donor and allowed successful pre-emptive live renal transplantation to take place.

## Abbreviations

ADTKD: Autosomal dominant tubulointerstitial kidney disease
AKI: Acute kidney injury
CKD: Chronic kidney disease
ESA: Erythropoiesis-stimulating agent
ESRD: End-stage renal disease.
